# Supplementary material for: Genetic Diversity of Avian Influenza Viruses Detected in Waterbirds in Northeast Italy Using Two Different Sampling Strategies
Source: Animals (Basel). 2024 Mar 27;14(7):1018. doi: 10.3390/ani14071018 (PMC11010841; doi:10.3390/ani14071018)
Supplement: Supplementary file 1 [file animals-14-01018-s001.zip › animals-2915964-supplementary.pdf]

Supplementary Materials

Table S1. GISAID Epi-Flu Database accession numbers of the PB2, PB1, PA, HA, NP, NA, M, NS coding sequences for the AIV isolates collected during autumn-winter 2021 in the Emilia Romagna region, Northern Italy.

Strain	Subtype	Location	Accession Number
A/teal/Italy/1821-10_22VIR4622-1/2021	H1N1	Buda, Bologna province	EPI3073287, EPI3073288, EPI3073289, EPI3073290, EPI3073291, EPI3073292, EPI3073293, EPI3073294
A/mallard/Italy/22VIR4203-2/2021	H5N3 LPAIV	Argenta, Ferrara province	EPI3075426, EPI3075427, EPI3075428, EPI3075429, EPI3075430, EPI3075431, EPI3075438, EPI3075467
A/teal/Italy/1856-7_22VIR4622-7/2021	H9N2	Buda, Bologna province	EPI3073297, EPI3073298, EPI3073299, EPI3073300, EPI3073301, EPI3073302, EPI3073303, EPI3073304
A/teal/Italy/1828-6_22VIR4622-5/2021	H9N2	Buda, Bologna province	EPI3075410, EPI3075411, EPI3075412, EPI3075413, EPI3075414, EPI3075415, EPI3075416, EPI3075417
A/teal/Italy/1821-14_22VIR4622-3/2021	H9N2	Buda, Bologna province	EPI3075418, EPI3075419, EPI3075420, EPI3075421, EPI3075422, EPI3075423, EPI3075424, EPI3075425

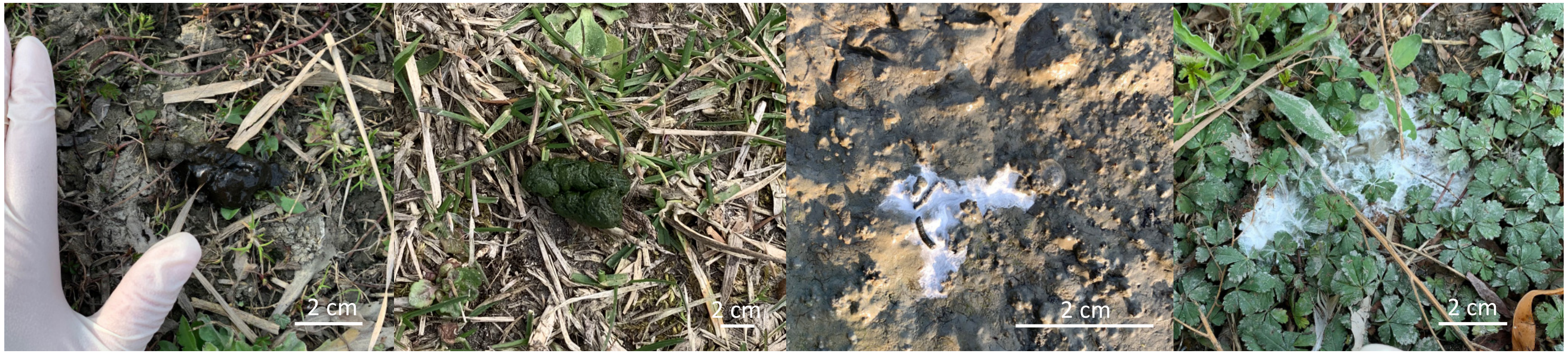

Figure S1. Avian faecal droppings from ducks (A), geese (B), shorebirds (C), herons (D). Specimens were identified through direct observations of Northern shoveler, greylag goose, common snipe, and grey heron, respectively, during sampling activities.

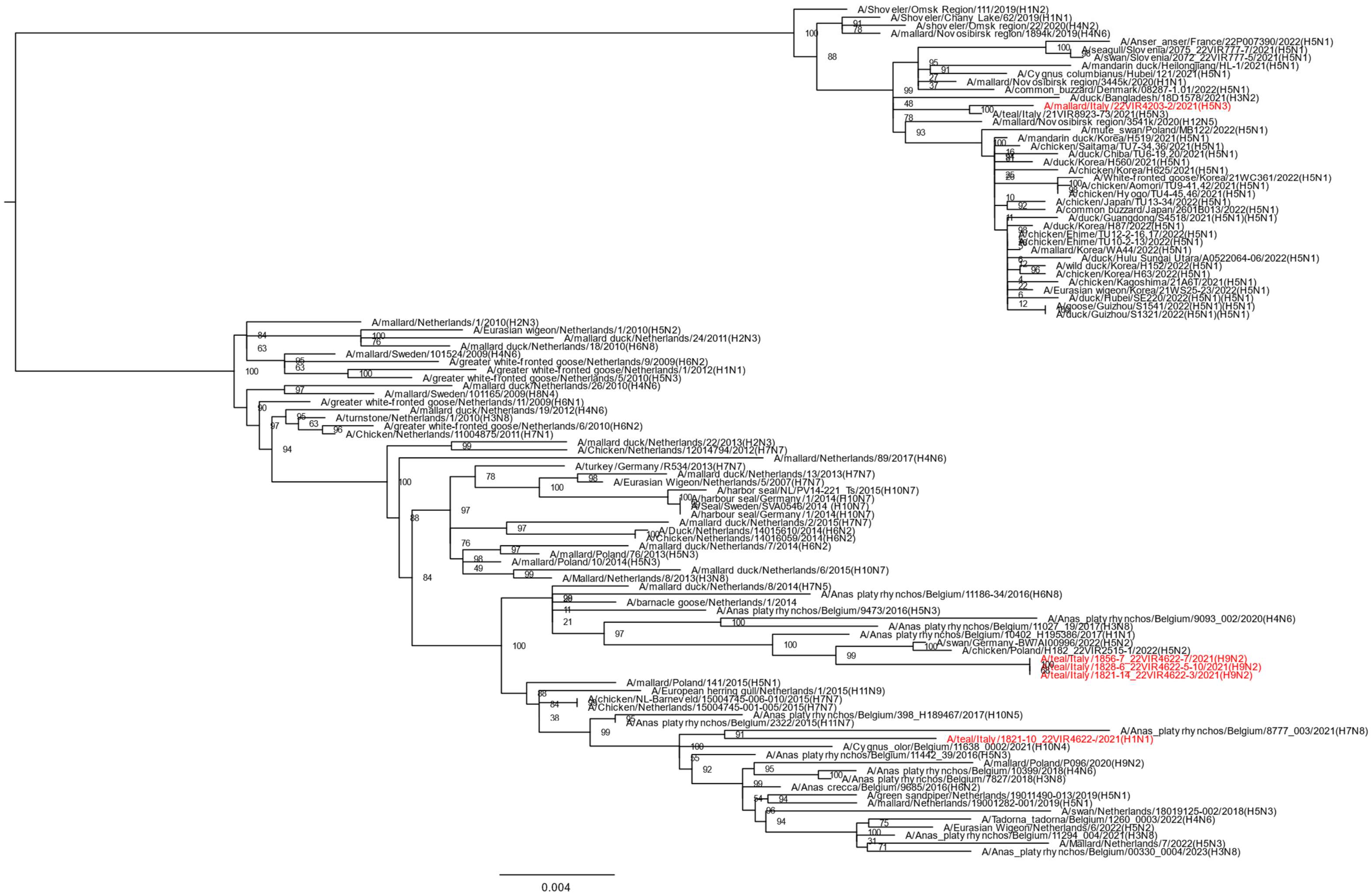

Figure S2. PB2 sequences phylogenetic maximum-likelihood reconstruction. The PB2 gene sequences of the isolates hereby obtained, namely A/teal/Italy/1821-10_22VIR4622-1/2021(H1N1), A/mallard/Italy/22VIR4203-2/2021 (H5N3) LPAI, A/teal/Italy/1856-7_22VIR4622-7/2021 (H9N2), A/teal/Italy/1828-6_22VIR4622-5/2021 (H9N2), A/teal/Italy/1821-14_22VIR4622-3/2021 (H9N2) are shown in red. The substitution model for the maximum likelihood phylogenetic reconstruction was TIM+F+G4.

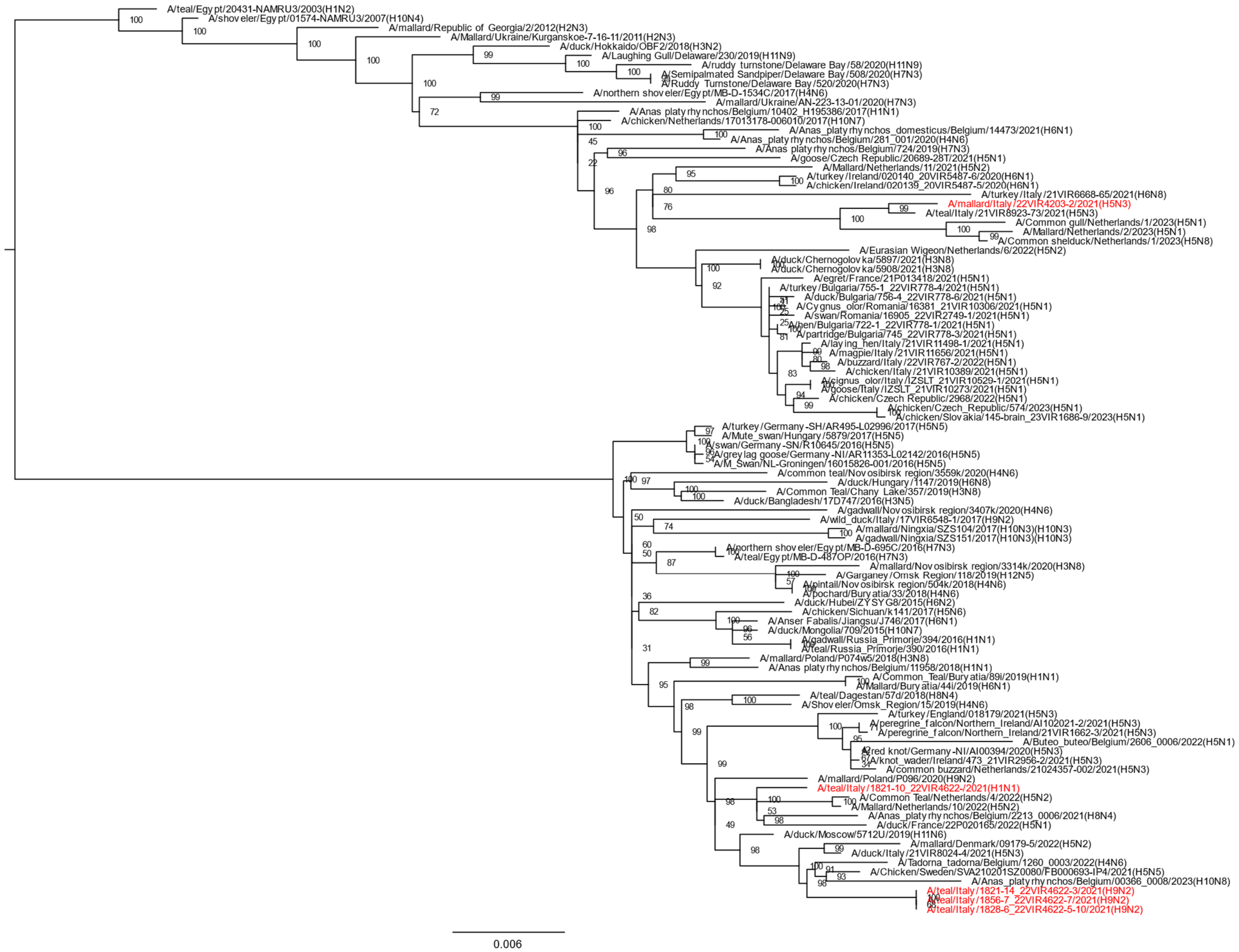

Figure S3. PB1 sequences phylogenetic maximum-likelihood reconstruction. The PB1 gene sequences of the isolates hereby obtained, namely A/teal/Italy/1821-10_22VIR4622-1/2021(H1N1), A/mallard/Italy/22VIR4203-2/2021 (H5N3) LPAI, A/teal/Italy/1856-7_22VIR4622-7/2021 (H9N2),

A/teal/Italy/1828-6_22VIR4622-5/2021 (H9N2), A/teal/Italy/1821-14_22VIR4622-3/2021 (H9N2) are shown in red. The substitution model for the maximum likelihood phylogenetic reconstruction was TIM+F+G4.

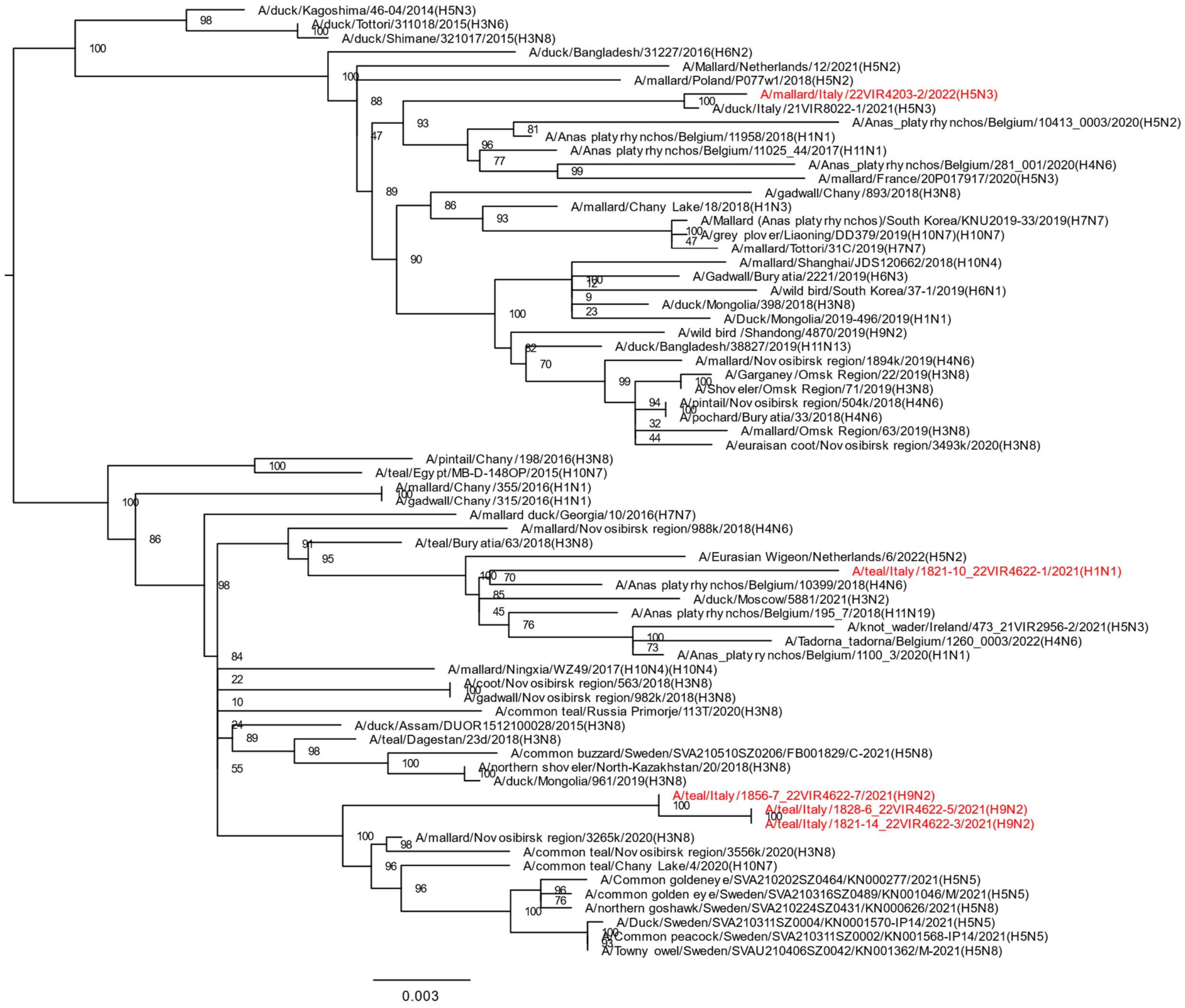

Figure S4. PA sequences phylogenetic maximum-likelihood reconstruction. The PA gene sequences of the isolates hereby obtained, namely A/teal/Italy/1821-10_22VIR4622-1/2021(H1N1), A/mallard/Italy/22VIR4203-2/2021 (H5N3) LPAI, A/teal/Italy/1856-7_22VIR4622-7/2021 (H9N2),

A/teal/Italy/1828-6_22VIR4622-5/2021 (H9N2), A/teal/Italy/1821-14_22VIR4622-3/2021 (H9N2) are shown in red. The substitution model for the maximum likelihood phylogenetic reconstruction was K3Pu+F+G4.

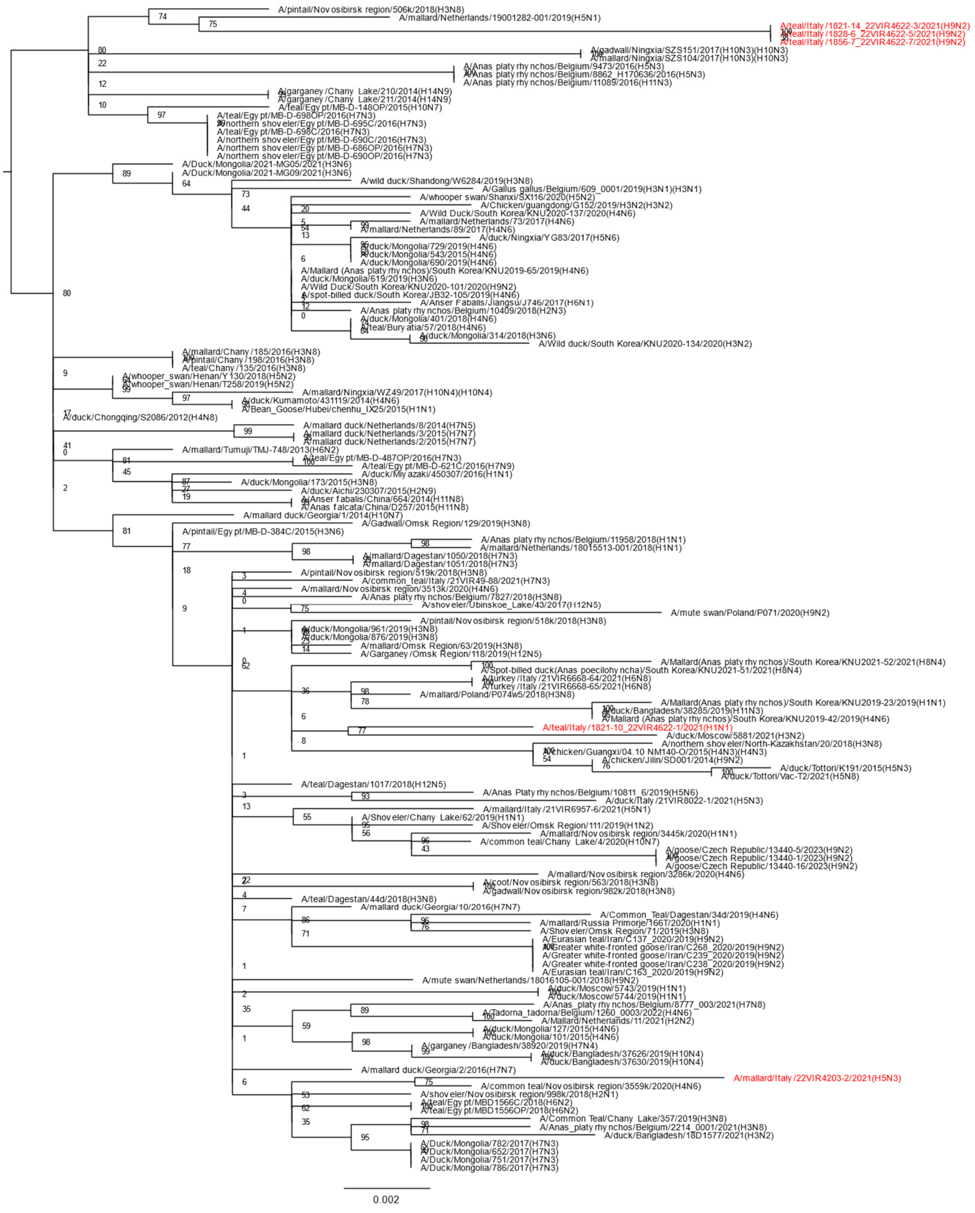

Figure S5. M sequences phylogenetic maximum-likelihood reconstruction. The M gene sequences of the isolates hereby obtained, namely A/teal/Italy/1821-10_22VIR4622-1/2021(H1N1), A/mallard/Italy/22VIR4203-2/2021 (H5N3) LPAI, A/teal/Italy/1856-7_22VIR4622-7/2021 (H9N2), A/teal/Italy/1828-6_22VIR4622-5/2021 (H9N2), A/teal/Italy/1821-14_22VIR4622-3/2021 (H9N2) are shown in red. The substitution model for the maximum likelihood phylogenetic reconstruction was K3P+I+G4.

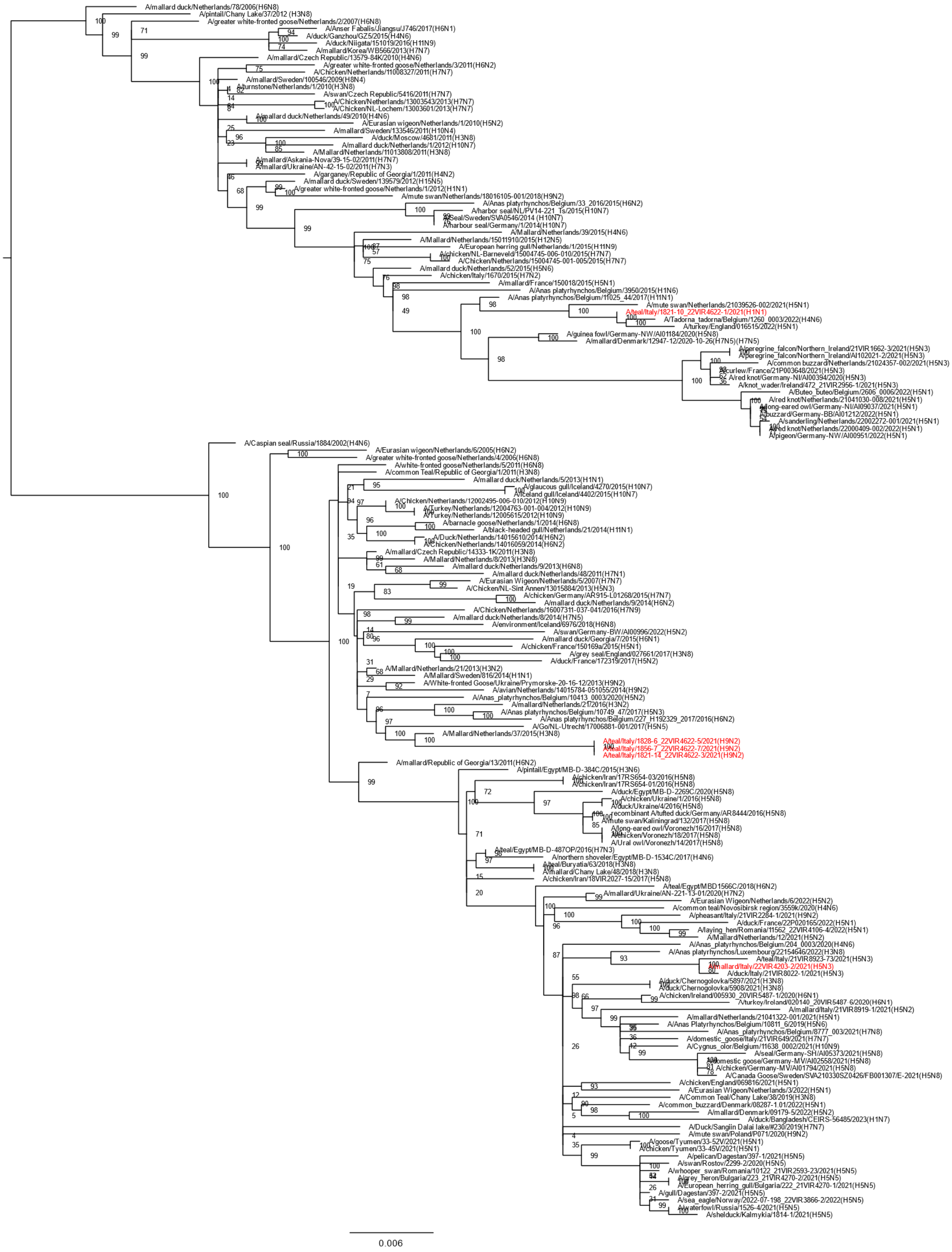

Figure S6. NP sequences phylogenetic maximum-likelihood reconstruction. The NP gene sequences of the isolates hereby obtained, namely A/teal/Italy/1821-10_22VIR4622-1/2021(H1N1), A/mallard/Italy/22VIR4203-2/2021 (H5N3) LPAI, A/teal/Italy/1856-7_22VIR4622-7/2021 (H9N2), A/teal/Italy/1828-6_22VIR4622-5/2021 (H9N2), A/teal/Italy/1821-14_22VIR4622-3/2021 (H9N2) are shown in red. The substitution model for the maximum likelihood phylogenetic reconstruction was K3Pu+F+I+G4.

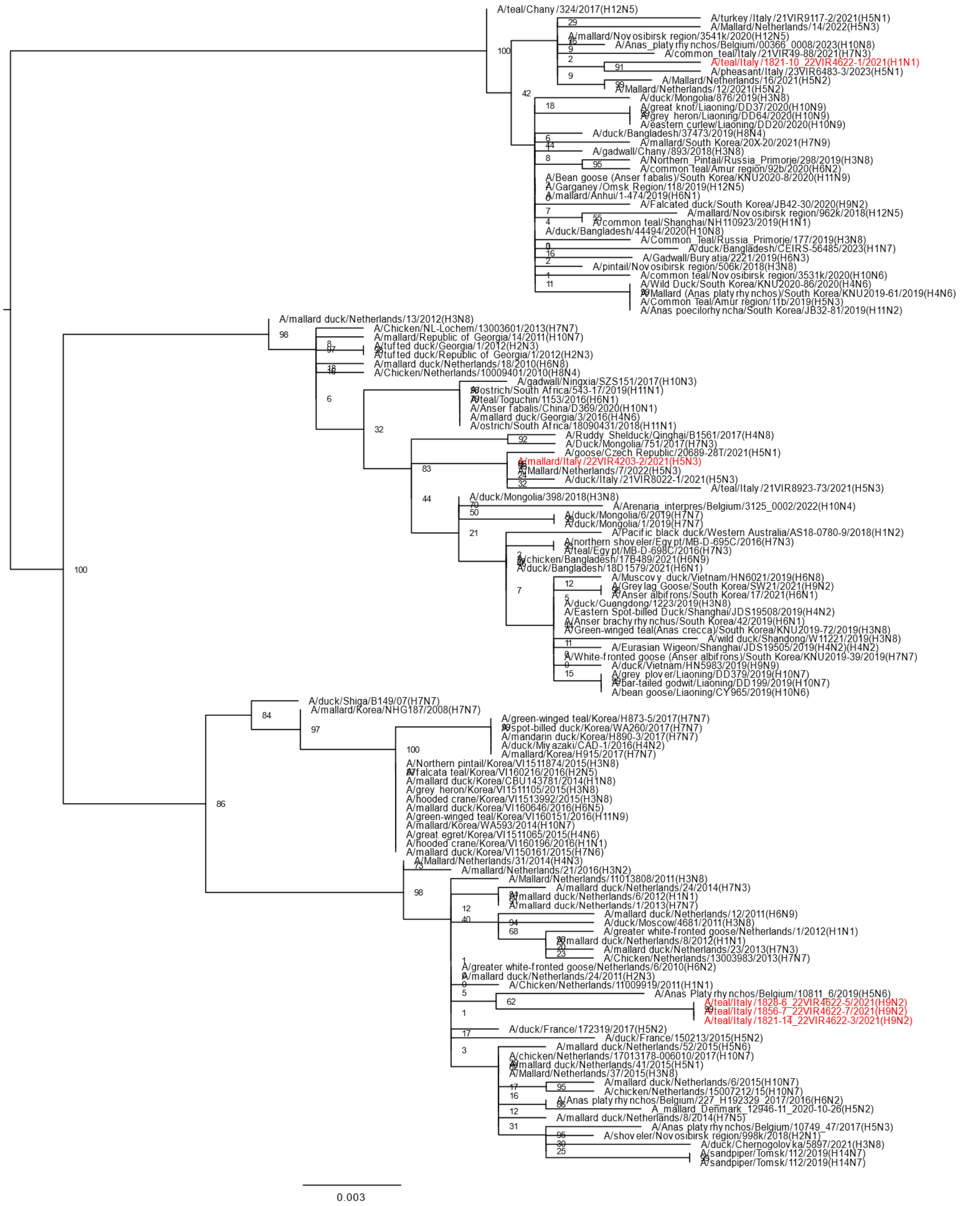

Figure S7. NS sequences phylogenetic maximum-likelihood reconstruction. The NS gene sequences of the isolates hereby obtained, namely A/teal/Italy/1821-10_22VIR4622-1/2021(H1N1), A/mallard/Italy/22VIR4203-2/2021 (H5N3) LPAI, A/teal/Italy/1856-7_22VIR4622-7/2021 (H9N2), A/teal/Italy/1828-6_22VIR4622-5/2021 (H9N2), A/teal/Italy/1821-14_22VIR4622-3/2021 (H9N2) are shown in red. The substitution model for the maximum likelihood phylogenetic reconstruction was TPM2u+F+I.
